# *Phytophthora* ×*stagnum* nothosp. nov., a New Hybrid from Irrigation Reservoirs at Ornamental Plant Nurseries in Virginia

**DOI:** 10.1371/journal.pone.0103450

**Published:** 2014-07-29

**Authors:** Xiao Yang, Patricia A. Richardson, Chuanxue Hong

**Affiliations:** Hampton Roads Agricultural Research and Extension Center, Virginia Tech, Virginia Beach, Virginia, United States of America; Agriculture and Agri-Food Canada, Canada

## Abstract

A novel *Phytophthora* species was frequently recovered from irrigation reservoirs at several ornamental plant production facilities in eastern Virginia. Initial sequencing of the internal transcribed spacer (ITS) region of this species generated unreadable sequences due to continual polymorphic positions. Cloning and sequencing the ITS region as well as sequencing the mitochondrially encoded cytochrome *c* oxidase 1 and beta-tubulin genes revealed that it is a hybrid between *P.* taxon PgChlamydo as its paternal parent and an unknown species genetically close to *P. mississippiae* as its maternal parent. This hybrid has some diagnostic morphological features of *P.* taxon PgChlamydo and *P. mississippiae*. It produces catenulate hyphal swellings, characteristic of *P. mississippiae*, and chlamydospores, typical of *P.* taxon PgChlamydo. It also produces both ornamented and relatively smooth-walled oogonia. Ornamented oogonia are another important diagnostic character of *P. mississippiae*. The relatively smooth-walled oogonia may be indicative of oogonial character of *P.* taxon PgChlamydo. The new hybrid is described here as *Phytophthora* ×*stagnum*.

## Introduction

The genus *Phytophthora* includes many agriculturally and ecologically important plant pathogens. It currently contains approximately 120 species [1]. These species were traditionally divided into six groups by morphological features [2]. They have been classified into 10 clades according to phylogenetic analyses of nuclear and mitochondrial sequences [3]–[8]. Members of this genus are capable of surviving in a variety of terrestrial and aquatic habitats [9]. However, species in certain clades or subclades are better adapted to specific ecosystems. For example, most clade 1 species such as *P. infestans* [9] and *P. hedraiandra* [10] appear as terrestrial pathogens which attack above-ground plant tissues, while many species in subclade 6b and clade 9 are often associated with aquatic environments such as irrigation reservoirs [11]–[17], rivers and riparian ecosystems [18], [19].

Even though *Phytophthora* species were among the earliest described plant pathogens, investigations into their interspecific hybridization were initiated only recently. One of the first studies describing this phenomenon was conducted in 1991 which revealed that some isolates initially assigned as *P. meadii* were actually polyploid and might be hybrids based on cytological evidence [20]. Thereafter, several artificial hybrids: *P. infestans*×*P. mirabilis*, *P. nicotianae*×*P. capsici*, *P. sojae*×*P. vignae*, and *P. capsici*×*P. tropicalis* have been produced by pairing in dual culture [21]–[23], zoospore fusion [24], [25], and nuclear transplantation [26]. In the meanwhile, eleven natural *Phytophthora* hybrids have been reported. These include *P.* ×*pelgrandis* (*P. nicotianae*×*P. cactorum*) [27]–[30], *P. alni* including three subspecies: *P. alni* subsp. *alni*, *P. alni* subsp. *uniformis* and *P. alni* subsp. *multiformis* [31]–[33], *P. andina* with *P. infestans* as one parent [34]–[36], *P.* ×*serendipita* (*P. cactorum*×*P. hedraiandra*) [29], [37], four hybrids in subclade 6b: *P. amnicola*×*P.* taxon PgChlamydo (A-PG), *P.* taxon PgChlamydo×*P. amnicola* (PG-A), *P. thermophila*×*P. amnicola* (T-A), and *P. thermophila*×*P.* taxon PgChlamydo (T-PG) [38], as well as three hybrids in subclade 8b: *P. porri*×*P.* taxon parsley, *P. porri*×a *P. primulae*-like species, and a third hybrid with two unknown species as parents [39]. It is interesting to note that parents of most individual hybrids belong to the same *Phytophthora* clade. The only inter-clade hybrid is *P. nicotianae* (clade 1)× *P. capsici* (clade 2), which was produced by zoospore fusion [24], [25] and nuclear transplantation [26].

A number of *Phytophthora* hybrids are emerging plant pathogens. By inheriting and recombining alleles or genes from both parents followed by rapid evolution [39]–[41], these hybrids have broader host ranges [39] and produce new virulence factors with higher aggressiveness, while overcoming weaknesses of their parental species. For example, *P. alni* and its variants are destructive pathogens that have killed more than 10,000 riparian *Alnus* trees in Europe in 1996 alone [31]. *Phytophthora* ×*pelgrandis* was found infecting plants in the genera of *Cyclamen*, *Eriobotrya*, *Lavandula*, *Lewisia*, *Pelargonium*, *Primula*, and *Spathiphyllum* in the Netherlands, Germany, Italy, Peru and Taiwan [27]–[30], [42], [43]. *Phytophthora* ×*serendipita* has been isolated from hosts in the genera of *Idesia*, *Penstemon*, *Allium*, *Rhododendron*, *Kalmia*, and *Dicentra* in Europe and the United States, while its parent *P. hedraiandra* only infects *Rhododendron* and *Viburnum* species, indicating this emerging hybrid pathogen has successfully utilized new habitats and adapted to novel hosts [29], [37]. *Phytophthora porri*×*P.* taxon parsley in subclade 8b has shown a similar expansion of host range including *Allium victorialis*, *Allium grayi*, *Pastinaca sativa*, *Chrysanthemum* species, and *Parthenium argentatum*, while its parents only infect leek and parsley [39]. Although their host ranges are unknown, the four subclade 6b hybrids A-PG, PG-A, T-A, and T-PG, which originated in Australia, have exploited new habitats in South Africa [38]. It must be noted that sexual reproduction of most *Phytophthora* hybrids is compromised due to their nature of allopolyploidy and resulting genetic incompatibility. Most *Phytophthora* hybrids are sterile, nonfunctional in meiosis, or produce numerous abortive oospores [29], [32], [38], [39].

Since 2005 we have obtained more than twenty isolates of a previously unknown *Phytophthora* species from irrigation systems. It has distinct morphology from all known species. Also, continual polymorphic sequences in the internal transcribed spacers (ITS) region of all isolates suggest that this is a *Phytophthora* hybrid. Here, we examine and describe its morphological, physiological and molecular characters and name this new hybrid as *Phytophthora* ×*stagnum* nothosp. nov.

## Materials and Methods

### Ethics statement

This study is part of a large collaborative project with several ornamental plant nurseries in Virginia from which isolates of *Phytophthora* ×*stagnum* were collected. Our field sampling did not involve endangered or protected species. No specific permission was required. Specific information about these properties is not disclosed to protect the businesses of these collaborating growers.

### Isolate collection and maintenance

*Phytophthora* ×*stagnum* isolates were recovered from irrigation runoff containment basins of several private ornamental plant nurseries in eastern Virginia, USA, by baiting with rhododendron leaves. Pure cultures were obtained by subculturing hyphal tips of colonies emerging from the edge of leaf baits followed by single-spore isolation [9]. They were maintained and routinely subcultured onto 20% clarified V8 juice agar (CV8A) in the present study. Agar blocks with actively growing cultures in CV8A were transferred into microtubes with sterile distilled water for long-term storage at 15°C. The holotype was deposited at the American Type Culture Collection (MYA-4926) in Manassas, Virginia.

### DNA extraction

Four representative isolates, 36H8, 36J7, 43F3, and 44F9, were grown in 20% clarified V8 broth at room temperature (*c*. 23°C) for 7 days to produce mycelial masses which were then dried and lysed using a FastPrep-24 system (MP Biomedicals, Santa Ana, CA, USA). DNA was extracted using the DNeasy Plant Mini kit (Qiagen, Valencia, CA, USA).

### Sequence analysis of the maternally-inherited *cox* 1 genes

To elucidate the maternal parent of *P.* ×*stagnum*, primers COXF4N and COXR4N [5] were used to amplify the maternal-inherited mitochondrial cytochrome *c* oxidase 1 (*cox* 1) gene. Sequences in both directions were visualized with Finch TV *v*. 1.4.0 (Geospiza Inc., Seattle, WA, USA), aligned using ClustalW and edited manually to correct obvious errors. The *cox* 1 sequences were aligned using MAFFT online version 7 [44] and the G-INS-I algorithm [45]. Maximum likelihood (ML) inference was carried out with MEGA5.1 [46] using the Tamura-Nei model [47] with 1,000 bootstrap replicates. *Pythium aphanidermatum* was used as an outgroup.

### Sequence analyses of ITS and beta-tubulin genes

To investigate the parentage of *P.* ×*stagnum*, cloned ITS region and the single-copy beta-tubulin genes were sequenced and analyzed.

PCR amplifications were performed using the forward primer ITS6 and reverse primer ITS4 [3] for the ITS region. Amplification products were cloned into a pGEM-T Easy Vector System, which was then transformed into *Escherichia coli* competent JM109 cells (Promega, Madison, WI, USA). The cells were plated on Luria-Bertani (LB) agar (Becton, Dickinson and Company, Sparks, MD, USA) amended with ampicillin and ChromoMax IPTG/X-Gal Solution (Fisher Scientific, USA) and incubated at 37°C. Transformed cells with recombinant plasmids were identified by blue-white screening, subcultured into 2-mL centrifuge tubes containing 1.5 mL LB broth using toothpicks, and incubated overnight at 37°C with moderate shaking. Plasmid DNA was extracted from the liquid cultures using the Alkaline Lysis with SDS: Minipreparation method [48]. The ITS primer pair 6F/4R was used to amplify the plasmid DNA. A total of 94 amplification products including 23, 23, 25, and 23 from isolates 36H8, 36J7, 43F3, and 44F9, respectively, were purified and sequenced at the University of Kentucky Advanced Genetic Technologies Center (Lexington, KY, USA) in both directions using the same ITS primer pair.

Primers Btub_F1 and Btub_R1 [4] were used to amplify the single-copy beta-tubulin gene. To analyze hybrid characteristic of *P.* ×*stagnum*, edited sequences were compared to those of putative parent species. Alignments were done with ClustalW.

### Colony morphology

To examine colony morphology, cultures of four representative isolates were grown on carrot agar (CA), CV8A, malt extract agar (MEA), and potato dextrose agar (PDA). Colony patterns were photographed after incubation for 10 days in the dark at 20°C.

### Cardinal temperatures

Representative isolates were examined for their cardinal temperatures on CA and CV8A. Agar blocks (5 mm in diameter) taken from actively-growing areas of 10-day old cultures were placed at the center of 10-cm Petri dishes with freshly made media. Triplicate dishes per isolate per temperature were placed in the dark at 5, 10, 15, 20, 25, 30, 35, and 40°C. Two perpendicular measurements of each colony were taken after 8 days. The cardinal temperature test was repeated once. Means of radial growth along with standard errors were plotted against temperature with the gplot package 2.11.0 [49] in R statistical software 2.15.0 [50]. Analysis of variance was also conducted with R to determine the differences in radial growth measurements between repeated experiments and among representative isolates.

### Morphology

Sporangia of *Phytophthora* ×*stagnum* were produced by transferring agar plugs (10×10 mm) from actively growing cultures on CV8A to Petri dishes containing non-sterile, soil water extract (SWE, 15 g of sandy loam soil/1 L water). Mature sporangia developed after incubating at room temperature under cool-white fluorescent light. Chlamydospores were produced in aged cultures in CV8A (after >30 days).

The mating type of representative isolates was determined in dual culture with an A1 or A2 tester of *P. cinnamomi* on CV8A. Selfed gametangia of *P.* ×*stagnum* were induced in polycarbonate membrane tests with an opposite mating type tester of *P. nicotianae* using hemp seed agar (HSA) [51], [52].

Asexual and sexual bodies were photographed with a Nikon Fujix Digital Camera HC-300Zi connected to a Nikon Labophot-2 microscope. More than 50 randomly selected mature sporangia per isolate, more than 30 chlamydospores and all observed gametangia were measured using Image-Pro Plus *v*. 5.1.2.53.

### Nomenclature

The electronic version of this article in Portable Document Format (PDF) in a work with an ISSN or ISBN will represent a published work according to the International Code of Nomenclature for algae, fungi, and plants, and hence the new names contained in the electronic publication of a PLOS ONE article are effectively published under that Code from the electronic edition alone, so there is no longer any need to provide printed copies.

In addition, new name contained in this work has been submitted to MycoBank from where it will be made available to the Global Names Index. The unique MycoBank number can be resolved and the associated information viewed through any standard web browser by appending the MycoBank number contained in this publication to the prefix http://www.mycobank.org/MB/. The online version of this work is archived and available from the following digital repositories: PubMed Central, LOCKSS.

## Results

### Sequence analysis of *cox* 1 gene

**Figure 1:**
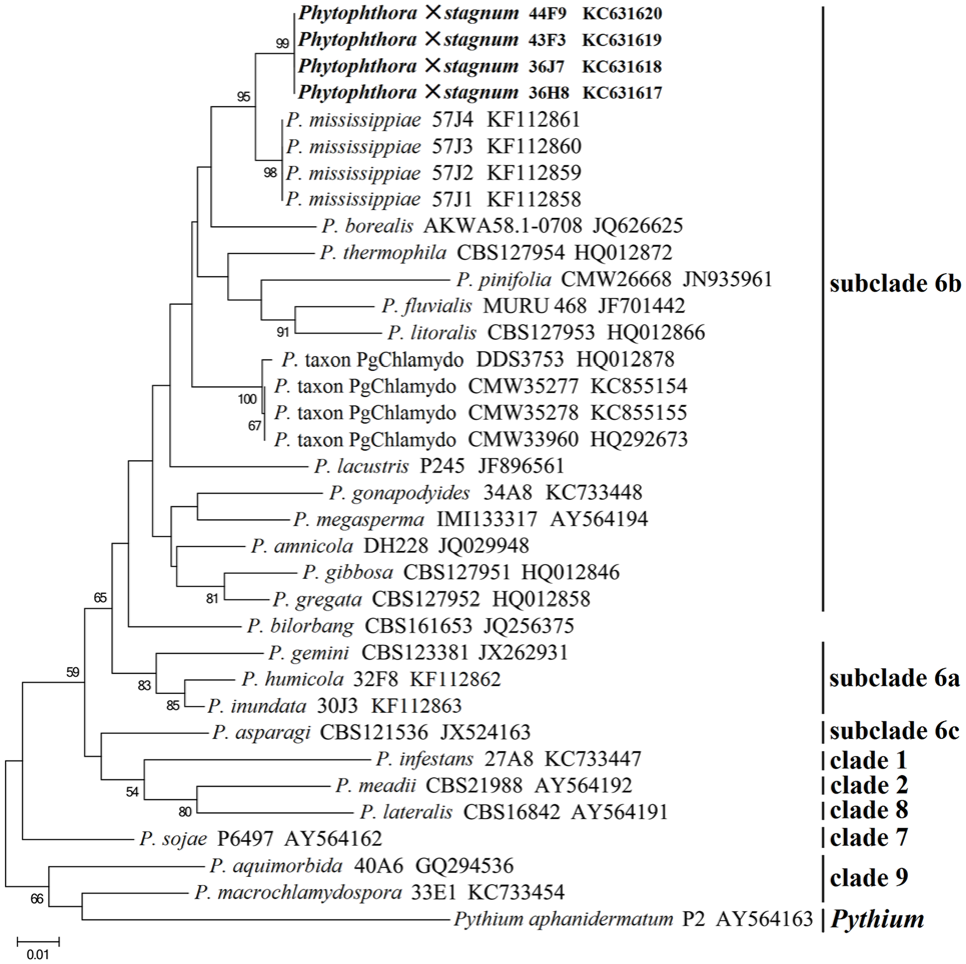
Maximum Likelihood phylogenetic tree based on mitochondrial *cox* 1 sequences of *Phytophthora* ×*stagnum* and representative species. Alignment was conducted with MAFFT version 7. Phylogenetic tree was generated in MEGA5. GenBank accession numbers of sequences are given following the species names and isolate codes. Bootstrap values are shown on branches (1,000 replicates; values <50% are not shown).

All four representative isolates of *P.* ×*stagnum* produced an identical 867-bp *cox* 1 sequence, which is distinct from those of all known *Phytophthora* species. This new species and *P. mississippiae* isolate 57J3 (GenBank Accession No. KF112860) differ by 18 bp in the alignment of *cox* 1 sequences. In the ML phylogenetic tree based on *cox* 1 sequences of *P.* ×*stagnum* and other selected species, *P.* ×*stagnum* isolates clustered in a distinct taxon which is closely related to *P. mississippiae* (Figure 1), indicating the maternal parent of *P.* ×*stagnum* is genetically close to *P. mississippiae*.

### Sequence analysis of ITS clones

**Figure 2:**
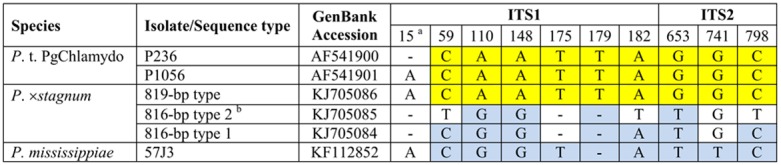
Internal transcribed spacer (ITS) sequence alignment of *Phytophthora* ×*stagnum*, *P. mississippiae* and *P.* taxon PgChlamydo. Position numbers are given based on the alignment. Yellow indicates sequences belong to *P.* taxon PgChlamydo authentic isolates. Blue indicates sequences belong to *P. mississippiae* type isolate 57J3. ^a^Position 15 is in the poly(A) region of ITS 1, which may contain sequencing errors. Thus, it is excluded from the analysis of hybridization. However, the indel of position 15 among three types of *P.* ×*stagnum* clones explains continual polymorphism and unreadable sequences of the ITS 1 regions amplified with the forward primer ITS6F in the initial sequencing before cloning. ^b^Type 1 and 2 occurred 21 and 10 times among 31 clones producing 816-bp sequences.

Among the 94 clones of the ITS region, 61 resulted in high-quality sequences. These included 16, 16, 16, and 13 from isolates 36H8, 36J7, 43F3, and 44F9, respectively. In the alignment of these 61 ITS sequences, 35 rare single-nucleotide polymorphism (SNP) sites occurred at low frequencies (∼1/61). These rare SNPs were mostly intraspecific polymorphisms of parent species of *P.* ×*stagnum*. We also observed six frequent SNPs (four in ITS1 region and two in ITS2 region) and three indels in the ITS1 region (Figure 2) at high frequencies (10–31/61).

These 61 clones can be generally grouped into three types by the six frequent SNPs and three indels in the ITS sequence. Thirty-one clones produced two types of 816-bp sequences while the other 30 clones produced an identical 819-bp sequence. According to the sequence alignment (Figure 2), the two 816-bp sequences are 99% identical to that of *P. mississippiae*, while the 819-bp sequence is ∼100% identical to those of *P.* taxon PgChlamydo in GenBank (www.ncbi.nlm.gov/genbank/). Clones of individual representative isolates produced all ITS sequence types.

### Sequence analysis of beta-tubulin gene

**Figure 3:**
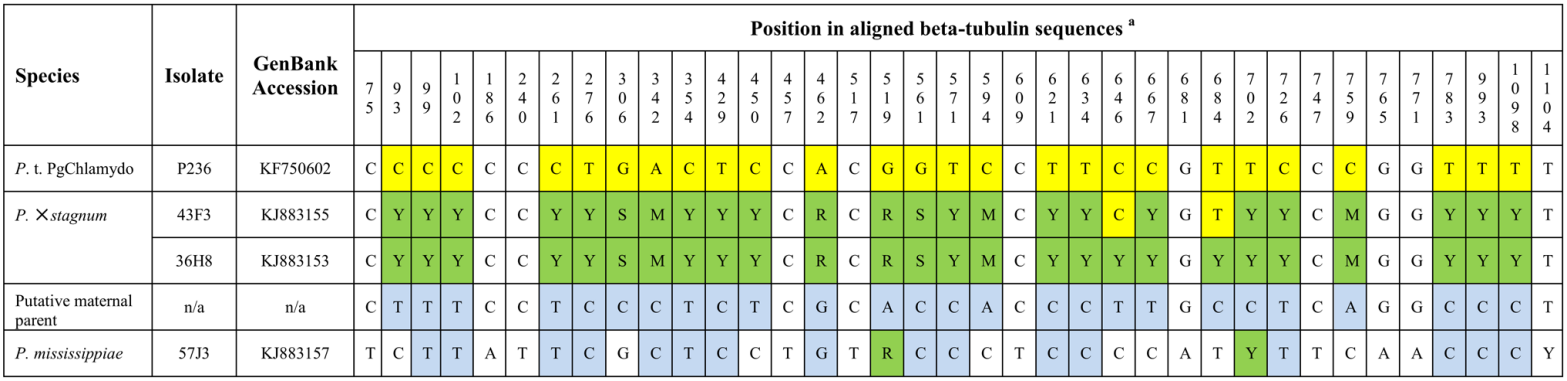
Beta-tubulin sequence alignment of *Phytophthora* ×*stagnum*, *P. mississippiae* and *P.* taxon PgChlamydo. Position numbers are given based on the alignment. Shading color: green indicates ambiguous sequences of polymorphic positions; yellow indicates sequences of the paternal parent *P.* taxon PgChlamydo; blue indicates sequences of the putative maternal parent. ^a^Y = T and C; S = G and C; M = A and C; R = G and A.

Isolates 36H8, 36J7, and 44F9 resulted in an identical 1124-bp beta-tubulin sequence with 26 polymorphic positions (Figure 3). Isolate 43F3 also produced a 1124-bp sequence with 24 of the same 26 polymorphic positions. In spite of the polymorphic positions, beta-tubulin sequences of *P.* ×*stagnum* are identical to that of *P.* taxon PgChlamydo and 11 bp different from that of *P. mississippiae* (Figure 3). Sequences of *P.* taxon PgChlamydo and *P. mississippiae* are distinct and both occur at 18 of the 26 polymorphic positions of *P.* ×*stagnum* containing ambiguous sequences such as positions 99, 102, and 261 (Figure 3). At the other eight polymorphic positions such as positions 93, 306, and 450, both species share the same sequences which also occur as one of the ambiguous polymorphic sequences of *P.* ×*stagnum* (Figure 3). Putative sequences of the maternal parent of *P.* ×*stagnum* are shown in Figure 3. The maternal parent is approximately 21 bp different from *P. mississippiae* in beta-tubulin sequence.

### Colony morphology

**Figure 4:**
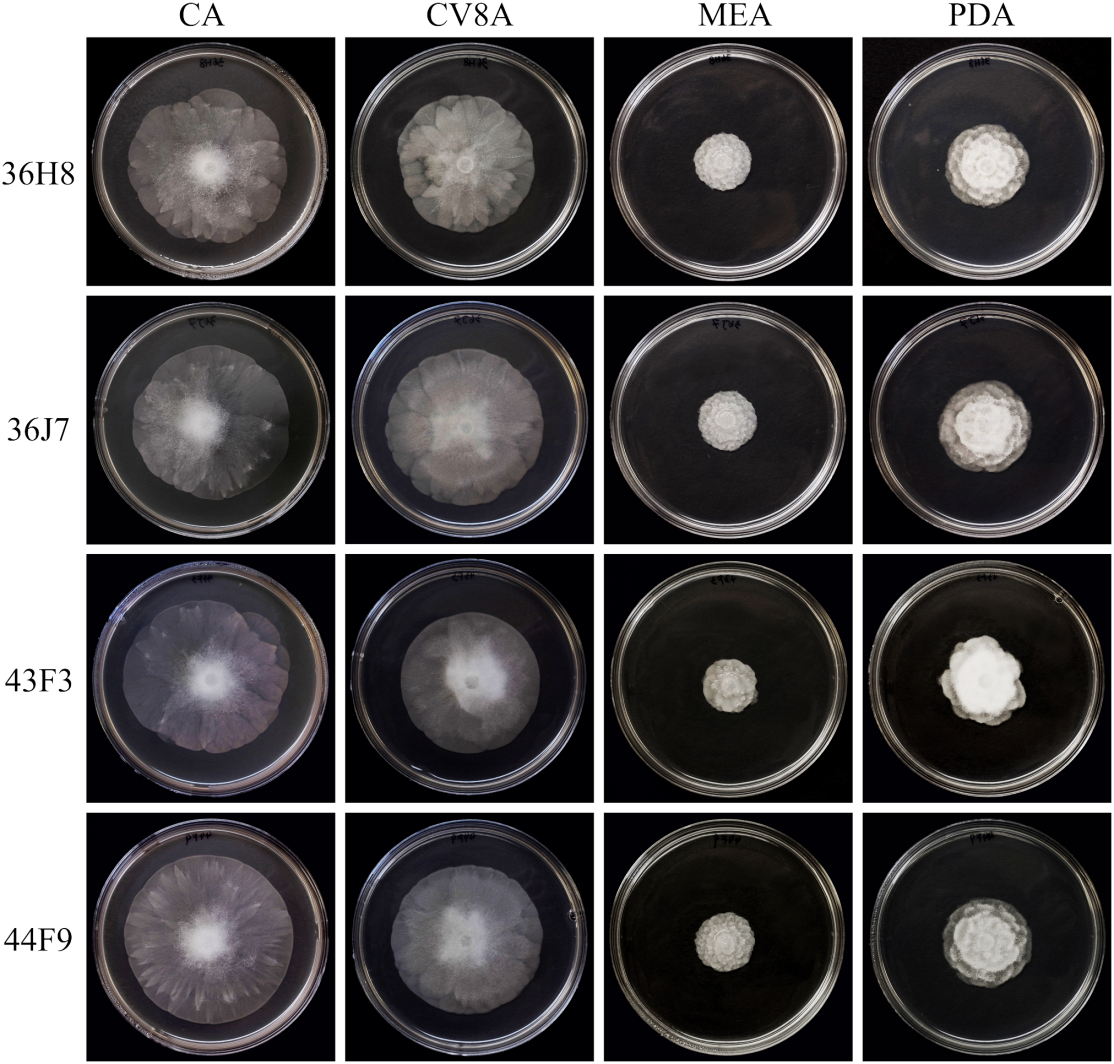
Colony morphology of *Phytophthora* ×*stagnum* representative isolates on various media incubated at 20°C for 10 days in the dark. CA = carrot agar, CV8A = 20% clarified V8 juice agar, MEA = malt extract agar, PDA = potato dextrose agar.

The four representative isolates had a similar growth pattern after 10-days incubation in the dark at 20°C (Figure 4). Colony pattern on CA and CV8A was stellate to radiate with a relatively smooth edge and abundant aerial mycelia at the center. Colony pattern on MEA and PDA was rosaceous except isolate 43F3, which produced a slightly cottony colony on PDA. Colony growth of all isolates was slowest on MEA among tested media.

### Cardinal temperatures for vegetative growth

**Figure 5:**
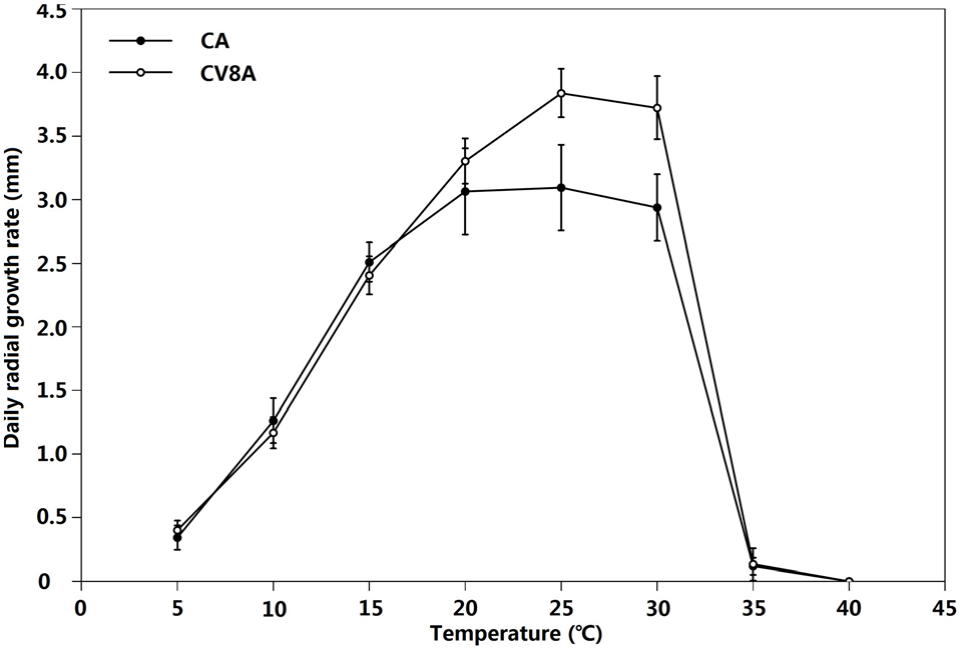
Average daily radial growth of *Phytophthora* ×*stagnum* representative isolates in carrot agar (CA) and 20% clarified V8 juice agar (CV8A) over an 8-days period.

Radial growth rates were similar among four representative isolates (*P* = 0.71) and between two cardinal temperature tests (*P* = 0.74). Thus, data from the repeated tests were pooled and averages were plotted against temperature (Figure 5). The optimum temperature for the vegetative growth of *P.* ×*stagnum* in both media was 25°C. It also grew well at 30°C on both media. Limited growth occurred at 5 and 35°C. No growth was observed at 40°C.

### Taxonomy

#### *Phytophthora ×stagnum*

**Figure 6:**
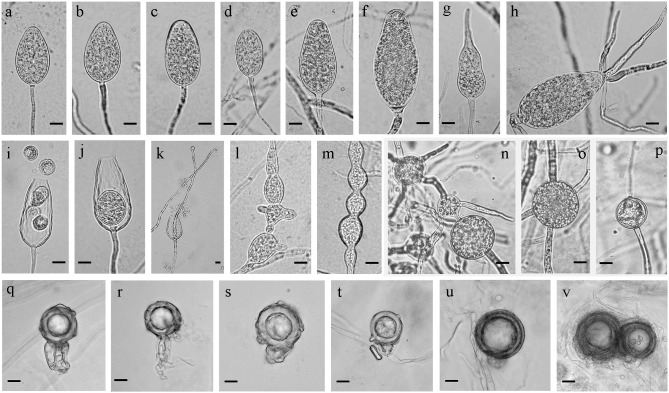
Morphology of *Phytophthora* ×*stagnum*. (a–g) Nonpapillate and noncaducous sporangia in various shapes; (a, b) Ovoid sporangia; (c, d) Ovoid to ellipsoid sporangia; (e, f) Obpyriform sporangia; (g) A germinated sporangium in distorted shape; (h) Direct germination of an obpyriform sporangium; (i) A sporangium releasing zoospores; (j) Nested internal proliferation; (k) Extended internal proliferation; (l) Hyphal swellings; (m) Catenulate hyphal swellings; (n) Thin-walled intercalary chlamydospores and hyphal swellings; (o) A thin-walled intercalary chlamydospore; (p) A thick-walled chlamydospore; (q–t) Ornamented, aborted oogonia produced by isolates 36H8 and 36J7; (q) An oogonium with an amphigynous antheridium; (r–t) Ornamented oogonia with distorted antheridia; (u, v) Relatively smooth-walled, darkly pigmented oogonia produced by isolates 43F3 and 44F9; (u) An oogonium containing a plerotic oospore with a globose antheridium; (v) Oogonia with abortive oospores. Bars = 10 µm.

X. Yang & C. X. Hong nothosp. nov. (Figure 6).

MycoBank: MB807978 [urn:lsid:mycobank.org: MB807978].

Sporangia were occasionally produced by aged cultures (>30 days) grown in CA and CV8A. Abundant sporangia were produced from fresh mycelial plugs submerged in 1.5% SWE within 10 hours. Sporangial shape varied from ovoid (Figures 6a, b) to ellipsoid (Figures 6c, d), obpyriform (Figures 6e, f) and distorted shapes (Figure 6g). Sporangia were terminal, nonpapillate and noncaducous. They ranged from 30.5 to 89.7 µm in length (average 54.3±11.0 µm) and 17.5 to 40.4 µm in width (average 30.3±3.9 µm). Direct germination of sporangia was frequently observed (Figures 6g, h). Nested and extended internal proliferations were common (Figures 6j, k). Hyphal swellings in irregular shapes were abundantly produced in both young and aged cultures (Figures 6l, m). Catenulate, globose hyphal swellings were frequently observed in aged cultures (Figure 6m). Intercalary chlamydospores were observed in aged cultures of all examined isolates (Figures 6n, o, p). They were mostly thin-walled (Figures 6n, o), rarely thick-walled (Figure 6p), and averaged 33.5±4.9 µm in diameter.

*Phytophthora* ×*stagnum* is heterothallic and all isolates examined are A1. They produced no sexual structure in single culture. Oogonia were produced in dual culture when each *P.* ×*stagnum* isolate was paired with an A2 tester of *P. cinnamomi*. In the polycarbonate test, a limited number of gametangia (∼40) were produced by the four isolates after being paired with an A2 mating type tester of *P. nicotianae* for more than 50 days. Two distinct groups of gametangia were observed. Isolates 36H8 and 36J7 mostly produced ornamented oogonia with characteristic protuberances (Figures 6q, r, s, t). These oogonia averaged 33.6±8.1 µm in diameter. Oogonial wall was pigmented golden at maturity. All observed ornamented oogonia aborted (Figures 6q, r, s, t). Antheridia were amphigynous, commonly distorted (Figures 6s, t). They averaged 19.4 µm in depth and 14.2 µm in width. Isolates 43F3 and 44F9 mostly produced oogonia with a relatively smooth surface (Figures 6u, v). These oogonia averaged 28.0±5.6 µm in diameter. The oogonial wall was darkly golden-brown. Plerotic oospores (Figure 6u) were also mostly aborted (Figure 6v). Antheridia were amphigynous, globose or distorted, and averaged 10.0 µm in depth and 12.3 µm in width (Figures 6u, v).

### Holotype

ATCC MYA-4926 (exo-type: 43F3), recovered from an irrigation runoff reservoir, Virginia, USA, January, 2007. Other representative isolates were recovered from the same location: isolates 36H8 and 36J7, recovered in March, 2007; 44F9, recovered in May, 2007.

### Etymology

‘*stagnum*’ refers to the irrigation reservoirs where this novel hybrid species was recovered.

## Discussion

Sequence analyses of the *cox* 1, ITS, and beta-tubulin genes have demonstrated that *Phytophthora* ×*stagnum* is a hybrid species with a species genetically close to *P. mississippiae* as its maternal and *P.* taxon PgChlamydo as its paternal parent. First, the mitochondrial *cox* 1 sequence of *P.* ×*stagnum* is mostly analogous to that of *P. mississippiae* (Figure 1), suggesting that its maternal parent is genetically close to *P. mississippiae*. Second, cloning of the ITS region of *P.* ×*stagnum* isolates consistently resulted in two types of 816-bp sequences and one type of 819-bp sequence. The 819-bp sequence is identical or only 1-bp different from those of authentic *P.* taxon PgChlamydo isolates [18]. The two types of 816-bp sequences only differ from that of the *P. mississippiae* type isolate [14] by 3 or 6 bp (Figure 2). Third, *P.* ×*stagnum* contains the beta-tubulin sequences of *P.* taxon PgChlamydo and *P. mississippiae* at 26 polymorphic positions (Figure 3). Its sequences at non-polymorphic positions are identical to that of *P.* taxon PgChlamydo and only ∼10 bp different from that of *P. mississippiae*. These results of ITS and beta-tubulin sequence analyses indicate that *P.* ×*stagnum* is a hybrid between *P.* taxon PgChlamydo and a species genetically close to *P. mississippiae*.

This hybrid species has diagnostic morphological and physiological characters of *P.* taxon PgChlamydo and *P. mississippiae*. For instance, *P.* ×*stagnum* is similar to *P.* taxon PgChlamydo in producing chlamydospores, which are not produced by *P. mississippiae* [14]. However, both *P.* ×*stagnum* and *P. mississippiae* produce abundant catenulate hyphal swellings (Figure 6m) in aged cultures, as well as nested or extended internal proliferations (Figures 6j, k). Also, both *P.* ×*stagnum* and *P. mississippiae* produce ornamented oogonia (Figures 6q–t). The relatively smooth-walled oogonia produced by *P.* ×*stagnum* (Figures 6u, v) may implicate the oogonial morphology of *P.* taxon PgChlamydo although it has not been reported. In addition, *P.* ×*stagnum* is similar to *P. mississippiae* in colony morphology and growth rate on CV8A [14]. Both species produce radiate to slightly petaloid colonies with a relatively smooth edge (Figure 4) and the fastest growth on CV8A occurs at 25°C (Figure 5). *Phytophthora* ×*stagnum* can be separated from both parents by its optimal growth temperature on CA at 25°C (Figure 5), while it occurs at 30°C for *P. mississippiae* [14] and about 28°C for *P.* taxon PgChlamydo [18].

Although we have identified the two parent species of *P.* ×*stagnum* by molecular and morphological evidences, the mechanism by which this subclade 6b hybrid was produced remains unknown. It seems likely that this new hybrid formed asexually. One major reason is that species in subclade 6b tend to be homothallic as exemplified by *P. gibbosa*, *P. gregata*, and *P. megasperma* [19], [53], or “sterile” with unknown sexual structures such as *P. amnicola*, *P. thermophila* and *P.* taxon PgChlamydo [18], [19], [54]. This tendency may be a result of their adaptation to aquatic habitats [18], [19], [38]. The four subclade 6b hybrid species reported in 2013, PG-A, A-PG, T-A, T-PG also produced no gametangia [38]. In this study, we only observed a limited number of sexual bodies of *P.* ×*stagnum* (∼40) in five polycarbonate-membrane tests. These results along with previous findings indicate that hybrids in subclade 6b were more than likely formed asexually via hyphal anastomosis or zoospore fusion. However, Nagel et al. [38] suggested that the conditions used in laboratory mating tests may be not conducive to the formation of sexual bodies of subclade 6b species, while suitable conditions may exist in natural environments [38]. The formation mechanism of sexual structures of these subclade 6b hybrid species warrants further investigations.

Aquatic environments are ideal for the development and survival of natural *Phytophthora* hybrids. Many known *Phytophthora* hybrids have close association with aquatic environments. Examples include the four subclade 6b hybrids recovered from river and riparian ecosystems [38]; *Phytophthora alni* and its variants associated with riparian *Alnus* trees [31], [32]; and *Phytophthora* ×*pelgrandis* initially recovered from horticultural plants grown in hydroponic systems [28]. The fact that most natural *Phytophthora* hybrid species were initially identified from aquatic environments is interesting. First, natural aquatic ecosystems such as rivers, streams, and riparian habitats provide ideal environments for many plant species to grow. Consequently, *Phytophthora* species from various plant hosts have greater chances to aggregate and subsequently form hybrids under suitable conditions, such as *P. alni* and subclade 6b hybrids described in 2013. Similarly, hundreds of ornamental plants are grown in nurseries using hydroponic or recycling irrigation systems which greatly increase the chance of close contact between species. *Phytophthora* ×*pelgrandis*, *P.* ×*serendipita*, and *P.* ×*stagnum* may have formed in these systems by mating or anastomosis [28]. Second, newly formed *Phytophthora* hybrids may have a better opportunity to survive and adapt to aquatic ecosystems that contain a diverse variety of plant species. Third, aquatic environments favor asexual reproduction via motile zoospores or chlamydospores. This may be important for species that are sterile or nonfunctional in sexual reproduction as are all known *Phytophthora* hybrids. Fourth, for the saprophytic *Phytophthora* species in subclade 6b including PG-A, A-PG, T-A, T-PG [18], [19], [38] as well as *P.* ×*stagnum* in this study, the abundant plant debris in aquatic environments provides ideal microhabitats and nutrient sources. Fifth, water also offers hybrids vehicles for mobility compared to terrestrial environments, which may allow them to migrate into new habitats. In summary, aquatic environments may provide favorable conditions for *Phytophthora* hybrids to form, survive and disseminate.

All four representative isolates of *Phytophthora* ×*stagnum* are genetically stable. They were routinely subcultured on artificial media during the experimental period (∼2 years) and did not revert to either parent type. Also, sequencing of the ITS region of representative isolates was conducted several times in three years (2008, 2012, and 2013), and all ITS sequences obtained displayed similar polymorphisms. In addition, isolates of *P.* ×*stagnum* have been continually recovered from the same irrigation reservoirs since 2005. These observations suggest that this new hybrid species is relatively stable in the laboratory and in nature, and may have adapted to the irrigation systems of the surveyed nurseries in eastern Virginia.

Similar to the other four *Phytophthora* hybrids in subclade 6b [38], the pathogenicity of *P.* ×*stagnum* is yet to be determined. No diseased plant samples associated with this novel hybrid species has been received in the Disease Clinic at Hampton Roads Agricultural Research and Extension Center in Virginia Beach, Virginia. Also, in a preliminary pathogenicity test, *P.* ×*stagnum* caused little if any dieback on rhododendron plants (data not shown). The low aggressiveness of *P.* ×*stagnum* may be inherited from its parent species. *Phytophthora* taxon PgChlamydo is considered as an opportunistic plant pathogen [18], although it has been found to cause leaf spot on nursery stocks in California [55]. The maternal parent species of *P.* ×*stagnum* is close to *P. mississippiae*, which has an unknown host range [14].

Origin of this novel hybrid is not known at this time. Although *P.* taxon PgChlamydo has been frequently recovered from the same irrigation reservoirs, the maternal parent of *P.* ×*stagnum* has never been isolated. This observation may suggest that the new hybrid had been introduced to these nurseries via incoming ornamental plant materials. Crop health risk posed by this new hybrid species has yet to be assessed.

## Supporting Information

Table S1 **Daily radial growth measurements of four** ***Phytophthora*** ×***stagnum*** **representative isolates**. Examined isolates were grown in carrot agar and 20% clarified V8 juice agar over an 8-days period in two cardinal temperature tests.
(CSV)

Table S2 **Morphological measurements of four** ***Phytophthora*** ×***stagnum*** **representative isolates.** These measurements include the size of sporangia, oogonia and antheridia.
(CSV)
